# Socioeconomic status and structural brain development

**DOI:** 10.3389/fnins.2014.00276

**Published:** 2014-09-04

**Authors:** Natalie H. Brito, Kimberly G. Noble

**Affiliations:** Department of Pediatrics, Gertrude H. Sergievsky Center, Columbia UniversityNew York, NY, USA

**Keywords:** socioeconomic status, brain development, structural imaging, environmental variation

## Abstract

Recent advances in neuroimaging methods have made accessible new ways of disentangling the complex interplay between genetic and environmental factors that influence structural brain development. In recent years, research investigating associations between socioeconomic status (SES) and brain development have found significant links between SES and changes in brain structure, especially in areas related to memory, executive control, and emotion. This review focuses on studies examining links between structural brain development and SES disparities of the magnitude typically found in developing countries. We highlight how highly correlated measures of SES are differentially related to structural changes within the brain.

## Introduction

Human development does not occur within a vacuum. The environmental contexts and social connections a person experiences throughout his or her lifetime significantly impact the development of both cognitive and social skills. The incorporation of neuroscience into topics more commonly associated with the social sciences, such as culture or **socioeconomic status (SES)**, has led to an increased understanding of the mechanisms that underlie development across the lifespan. However, more research is necessary to disentangle the complexities surrounding early environmental variation and neural development. This review highlights studies examining links between structural brain development and SES disparities of the magnitude typically found in developing countries. We do not include studies examining children who have experienced extreme forms of early adversity, such as institutionalization or severe abuse. We also limit this review to findings concerning socioeconomic disparities in brain structure, as opposed to brain function.

KEY CONCEPT 1. Socioeconomic status (SES) Refers to an individual's access to economic and social resources, as well as the benefits and social standing that come from these resources. Most often measured by educational attainment, income, or occupation.

SES is a multidimensional construct, combining objective factors such as an individual's (or parent's) education, occupation, and income (McLoyd, 1998). Neighborhood SES is also often considered (Leventhal and Brooks-Gunn, 2000), as are subjective measures of social status (Adler et al., 2000). In 2012, 46.5 million people in the United States (15%) lived below the official **poverty** line (United States Census Bureau, 2012) and numerous studies have reported socioeconomic disparities profoundly affecting physical health, mental well-being, and cognitive development (Anderson and Armstead, 1995; Brooks-Gunn and Duncan, 1997; McLoyd, 1998; Evans, 2006). In turn, SES accounts for approximately 20% of the variance in childhood IQ (Gottfried et al., 2003) and it has been estimated that by age five, chronic poverty is associated with a 6- to 13-point IQ reduction (Brooks-Gunn and Duncan, 1997; Smith et al., 1997). Disparities in cognitive development outweigh disparities in physical health, possibly contributing to the propagation of poverty across generations (Duncan et al., 1998).

KEY CONCEPT 2. Poverty Comparison of a household's income with a threshold level of income that varies with family size and inflation. Households below the poverty threshold are considered “poor.” Households above this threshold are considered “not poor” even if the amount of money between “poor” and “not poor” is diminutive. Poverty guideline for a family of four in 2014 is $23,850.

Evidence suggests multiple possible, and non-mutually-exclusive, explanations for these findings. Socioeconomically disadvantaged children tend to experience less linguistic, social, and cognitive stimulation from their caregivers and home environments than children from higher SES homes (Hart and Risley, 1995; Bradley et al., 2001; Bradley and Corwyn, 2002; Rowe and Goldin-Meadow, 2009). Additionally, individuals from lower SES homes report more stressful events during their lifetime, and the biological response to stressors has been hypothesized as one of the underlying mechanisms for health and cognitive disparities in relation to SES (Anderson and Armstead, 1995; Hackman and Farah, 2009; Noble et al., 2012a).

**Figure 1:**
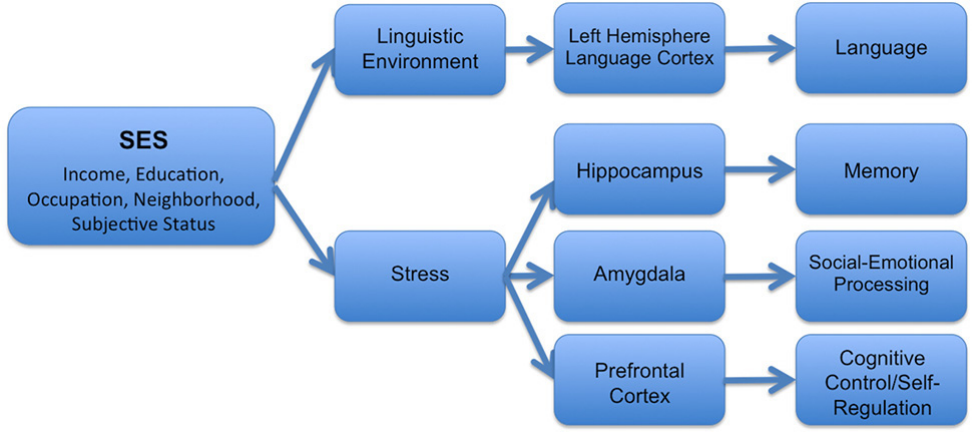
**Hypothesized mechanisms by which SES operates to influence structural and functional brain development**.

In turn, these experiential differences are likely to have relatively specific downstream effects on particular brain structures (see Figure 1 for one theoretical model). For example, disparities in the quantity and quality of linguistic stimulation in the home have been associated with developmental differences in language-supporting cortical regions in the left hemisphere (Kuhl et al., 2003; Conboy and Kuhl, 2007; Kuhl, 2007). In contrast, the experience of stress has important negative effects on the hippocampus (Buss et al., 2007; McEwen and Gianaros, 2010; Tottenham and Sheridan, 2010), the amygdala (McEwen and Gianaros, 2010; Tottenham and Sheridan, 2010), and areas of the prefrontal cortex (Liston et al., 2009; McEwen and Gianaros, 2010)—structures which are linked together anatomically and functionally (McEwen and Gianaros, 2010). As discussed below, different components of SES may differentially relate to these varying experiences, and thus may have varying associations with particular structures across the brain.

Measures of parental SES are often used as indicators of children's family or home conditions, but these distal measures may not fully account for children's experiences. For example, while a parent may be highly educated, unforeseen circumstances, such as a recession, may cause short- or long-term unemployment and inadequate income, leading to reduced resources and increased family stress experienced by the child. Studies examining an individual's own SES may more accurately represent the individual's current experience during adulthood, but may possibly discount the environmental experiences that shaped neural development as a child. Some studies have included measures of both childhood and adult SES (see Table 1), attempting to obtain a complete measure of SES development, but retrospective SES relies on the individual's memory of past events, and therefore may be biased. Overall, accurate and complete measures of SES are often difficult to obtain and these complications render it difficult to disentangle precise associations between specific socioeconomic indicators and outcomes of interest. Despite this, even approximate assessments of SES have, across multiple independent laboratories, been shown to predict clinically and statistically significant differences in brain structure and function, signifying the prominent association between environmental factors and brain development.

**Table 1:** **Studies reporting on associations between SES and structural brain development**.

	**Study**	**Participants**	**SES measures**	**Areas of the brain**	**Morphometry analysis**	**Main findings**
Children	Eckert et al., 2001	10–12 years old	**Participation in a federally subsidized school lunch program**.	Whole-brain and ROIs^a^ in planum temporale and central sulcus	MRI: cerebral volume (PV-wave and manual tracing); surface areas of ROIs (manual tracing)	Children who participated and children who did not participate in a federally subsidized school lunch program showed similar correlations between planum temporale asymmetry and phonological skill, although phonological skill was lower in the lower-SES group.
		*M* = 11.4 years	Low-income families had annual incomes less than $14,597			
		*N* = 39				
	Hanson et al., 2013	0–5 years old	**Family income**	Whole-brain and ROIs in frontal, parietal, temporal, and occipital lobes	MRI: cerebral volume; gray and white matter volumes in ROIs (Expectation–Maximization algorithm)	Children from lower income families had lower total gray matter volumes, and frontal and parietal volumes. No differences were found for total cerebral volume or parietal and temporal lobes. Children from lower income families showed reduced total gray matter trajectory.
		*M* = 13.5 months (first scan)	*Mode* = $50k to $75k			
			*Range* = $0 to > $100k			
		*N* = 77	**SES categories**			
			Low-SES (= or < 200% of FPL), Moderate-SES (between 200 and 400% of FPL), High-SES (greater than 400% of FPL)			
	Jednoróg et al., 2012	8–10 years old	**Hollingshead 2-factor index**	Whole-brain and ROIs in hippocampi, middle temporal gyri, left fusiform, right and inferior occipito-temporal gyri. Overall white matter microstructure	MRI: VBM^b^—total brain volume and gray matter volumes in ROIs (SPM8); SBM^c^—intracranial volume, hemispheric thickness, total surface area, and gray matter surface area, thickness, and volumes in ROIs (FreeSurfer) DTI^d^: Fractional anisotropy (BrainVISA and FSL)	Hollingshead Index positively correlated with gray matter volumes in hippocampi, parahippocampal, gyri, middle temporal gyri, insula, left fusiform gyrus, right inferior occipito-temporal region, and left superior/middle frontal gyrus. Hollingshead values not significantly correlated with white matter microstructure.
		*M* = 9.6 years	(maternal education and maternal occupation)			
		*N* = 23				
			*Mean* = 44, *SD* = 28			
			*Range* = 84–11			
			(Low- to high-SES families)			
	Luby et al., 2013	6–12 years old	**Family Income-to-Needs (ITN)^e^**	Whole-brain and ROIs in hippocampus and amygdala	MRI: cerebral volume; gray and white matter volumes in ROIs (FreeSurfer)	Family ITN positively correlated with total white and gray matter volumes as well as hippocampal and amygdala volumes. Effects of poverty on hippocampal volume were mediated by caregiving and stressful life events, but not parental education.
		*M* = 9.8 years	*Mean* = 2.14, *SD* = 1.27			
		*N* = 145	*Range* = 0–4.74			
			**Parental education**			
			*Mode* = Some college (38%)			
			*Range* = Less than HS to graduate degree			
	Raizada et al., 2008	5-year-olds	**Hollingshead 4-factor index**	Left inferior frontal gyrus	MRI: gray and white matter volume in ROI (SPM5)	Hollingshead Index was marginally positively correlated with both gray and white matter volumes in the left inferior frontal gyrus.
		*M* = 5.3 years	(Marital status, employment, educational attainment, and occupational prestige)			
		*N* = 14				
			*Range* = 31.5–66			
			(Middle- to high-SES families)			
Children and Adolescents	Hanson et al., 2011	4–18 years old	**Family income**	Whole-brain and ROIs in hippocampi and amygdalae	MRI: VBM- total brain volume and gray matter volumes in ROIs (DARTEL and SPM8)	Family income was positively correlated with hippocampal volume. No association between income and amygdala volumes. Positive correlation between paternal ED, but not maternal ED, and total and right hippocampal volumes. No relationship between income and cerebral volume.
		*M* = 11.2 years	*Range* = Less than $5k—more than $100k			
		*N* = 317				
			*Mode* = $75k–$100k (28%)			
			**Parental education**			
			*Range* = Less than HS to graduate degree			
			*Mode* = College (27%)			
	Lange et al., 2010	4–18 years old	**Family income**	Whole-brain and ROIs in intracranial cavity, cerebellum, brainstem, thalamus, caudate nucleus, putamen, globus pallidus, and frontal, temporal, parietal, and occipital lobes	MRI: total brain volume (sum of gray and white matter volumes in ROIs plus cerebrospinal fluid); gray and white matter volumes in ROIs (automated tissue segmentation algorithm)	Parental education levels were not correlated with brain volumes. Both family income and parental education were related to full scale IQ. Positive correlation between full scale IQ and cerebral volume. Total or regional brain volumes do not mediate association between parental education and IQ in children.
		*M* = 10.9 years	*Mean* = 73, 047, *SD* = 1816			
		*N* = 285	Less than $50K (27%), $50k–$100k (50%), greater than $100k (23%)			
			**Parental education**			
			*Range* = HS to graduate degree			
			*Mean* = 73, 047, *SD* = 1816			
			*Modes* = College (31%) and Graduate School (31%)			
	Brain Development Cooperative Group, 2012	4–18 years old	**Family income**	Whole-brain and ROIs in intracranial cavity, cerebellum, brainstem, thalamus, caudate nucleus, putamen, globus pallidus, and frontal, temporal, parietal, and occipital lobes	MRI: total brain volume (sum of gray and white matter volumes in ROIs plus cerebrospinal fluid); gray and white matter volumes in ROIs (“mni_autoreg” software package and automatic nonlinear image matching and anatomical labeling)	Family income and parental education levels were not associated with any regional brain volume.
		*M* = 10.9 years	*Mean* = 72, 458, *SD* = 31,695			
		*N* = 325	**Parental education**			
			*Modes* = College (31%) and graduate school (31%)			
			*Range* = HS to graduate degree			
	Lawson et al., 2013	4–18 years old	**Family income**	Frontal gyri (superior, middle and inferior), anterior cingulate gyri, and orbitofrontal gyri	MRI: cortical thickness (ANTS and DiReCT)	Parental education, but not family income, predicted increased cortical thickness in the left superior frontal gyrus and right anterior cingulate gyrus. No parental education by age interactions.
		*M* = 11.5 years	*Mode* = $75k–$100k (27%)			
		*N* = 283	*Range* = Less than $5k–$150k			
			**Parental education**			
			*Mean* = 7.53, *SD* = 2.31			
			*Range* = 2–12			
	Noble et al., 2012a	5–17 years old	**Income-to-Needs (ITN)**	Left temporal gyrus (superior, middle, and inferior), left fusiform gyrus, hippocampi, amygdalae, and anterior cingulate cortex	MRI: gray and white matter volumes in ROIs (FreeSurfer)	Parental education was negatively correlated with amygdala volume. No correlation between ITN and amygdala volume. ITN was positively correlated with hippocampal volume, but no correlation between parental education and hippocampal volume. Education by age interaction observed in left superior temporal gyrus and left inferior frontal gyrus.
		*M* = 11.4 years	*Mean* = 3.3, *SD* = 1.9			
		*N* = 60	*Range* = 0.23–6.7			
			**Parental education**			
			*Mean* = 15.1, *SD* = 2.7			
			*Range* = 8–21 years			
Adults	Butterworth et al., 2012	44–48 years old	**Experience of financial hardship over past year**	Amygdala and hippocampus	MRI: gray and white matter volumes in ROIs (FreeSurfer)	Experience of current financial hardship was correlated with smaller hippocampus and amygdala. Childhood poverty was not associated with either brain structure.
		*M* = 46.7 years				
		*N* = 403	4 dichotomous variables: pawned or sold something, went without meals, unable to heat home, or asked for help from welfare/community organizations			
			**Childhood poverty** (y/n)			
	Cavanagh et al., 2013	36–65 years old	**Early life SES (ESES)**	Cerebellum	MRI: cerebellar gray matter volume (FreeSurfer)	Both early life and current SES predicted cerebellar gray matter volume. Current SES explained significant additional variance to early life SES, but not vice-versa. Lower SES was associated with smaller cerebellar gray matter volumes.
		*M* = 50.94 years	(Number of siblings, people per room, paternal social class, parental housing tenure, and use of a car by family)			
		*N* = 42				
			**Current SES (CSES)**			
			(Current income, current social class, current housing tenure)			
	Chiang et al., 2011	18–29 years old	**Adult occupation (Australian socioeconomic index: SEI)**	Overall white matter microstructure	DTI: fractional anisotropy (FSL)	No main effect of SEI on white matter microstructure, but interaction between SEI and genetic components that affect white matter integrity. Higher SEI participants had higher heritability in the thalamus, left middle temporal gyrus, and callosal splenium. Lower SEI participants had higher heritability in the anterior corona radiate.
		*M* = 23.7 years				
		*N* = 499	*Median* = 67.5			
			*25th Percentile* = 39.7			
			*75th Percentile* = 83.8			
	Gianaros et al., 2007	31–54 years old	**Subjective social status (SSS)**	Anterior cingulate cortex, amygdala and hippocampus	MRI: VBM—total brain volume and gray matter volumes in ROIs (SPM2 and Matlab)	Lower subjective social status was associated with reduced gray matter volume in the perigenual area of the anterior cingulate cortex, but not anterior cingulate cortex, hippocampus, or amygdala. No associations between brain structures and educational attainment, income, personal, or community SES measures.
		*M* = 44.7 years	**Education**			
		*N* = 100	*Mode:* College (47%)			
			*Range* = Less than HS to PhD			
			**Income**			
			*Mode*: $50–65k (25%) and greater than $80k (25%)			
			**Personal SES** = composite of education and income			
			**Community SES** = zip code			
	Gianaros et al., 2012	30–50 years old	**Educational attainment**	Overall white matter microstructure	DTI: fractional anisotropy (FSL)	Individuals higher in education, earning higher incomes, and living in more advantaged communities demonstrated increases in white matter integrity and decreases in radial diffusivity.
		*M* = 40.7 years	*M* = 17.17, *SD* = 3.2			
		*N* = 155	*Range* = 11–24 years			
			**Income** Community SES			
	Krishnadas et al., 2013	35–64 years old	**Neighborhood SES**	Overall brain network structure and cortical thickness	MRI: cortical thickness (FreeSurfer)	Controlling for age and alcohol use, compared to the least deprived (LD) group the most deprived (MD) had significant cortical thinning in bilateral perisylvian cortices.
		*M* = 51 years	Scottish index of multiple deprivation (SIMD)			
		*N* = 42				
	Liu et al., 2012	67–79 years old	**Education**	Temporal pole, transverse temporal gyrus, and isthmus of cingulate cortex	MRI: volumes in 15 ROIs and cortical thickness in 33 ROIs (FreeSurfer)	Participants with higher levels of education had significantly larger temporal pole, transverse temporal gyrus, and isthmus of cingulate cortex.
		*M* = 73 years	*M* = 11 years, *SD* = 2.5			
		*N* = 113	*Range* = 6–16 years			
	Noble et al., 2012b	17–87 years old	**Educational attainment**	Amygdala and hippocampus	MRI: amygdala and hippocampal volumes (FreeSurfer)	Education by age interaction found in the hippocampus, such that the volumetric reduction seen at older ages was more pronounced among less educated individuals, and was buffered among more highly educated individuals. No main effects of education or age by education interactions found for amygdala volumes.
		*M* = 39.7 years	High school or less (32%)			
		*N* = 275	Some college (30%)			
			College and graduate degree (38%)			
	Noble et al., 2013	17–23 years old	**Educational attainment**	White matter microstructure (ROIs: superior longitudinal fasciculus, cingulum bundle, anterior coronal radiata)	DTI: fractional anisotropy (fMRIB Diffusion Toolbox and FNIRT)	Educational attainment significantly correlated with white matter microstructure in the superior longitudinal fasciculus and cingulum bundle (controlling for age).
		*M* = 20.1 years	*Mean* = 14.1, *SD* = 1.8			
		*N* = 47	*Range* = 11–18 years			
	Piras et al., 2011	18–65 years old	**Educational attainment**	Thalamus, caudate nucleus, putamen, globus palidus, hippocampus, and amygdala	MRI: Gray and white matter volumes in ROIs DTI: fractional anisotropy and mean diffusivity (FSL)	Educational attainment negatively correlated with microstructural changes in both left and right hippocampi (controlling for age).
		*M* = 40.35 years	*M* = 14.5, *SD* = 3.3			
		*N* = 150	*Range* = 5–21 years			
	Staff et al., 2012	Older adults	**Educational attainment**	Hippocampus	MRI: hippocampal volume (FreeSurfer)	Childhood SES (latent factor including paternal occupation and childhood home environment) positively correlated with hippocampal volume after adjusting for mental ability (at age 11), adult SES (self-occupation and current neighborhood environment) and educational attainment.
		*M* = 68.7 years	**Paternal occupation**			
		*N* = 235	Retrospective at age 11			
			**Self-occupation**			
			*Range* = 1–9			
			**Current neighborhood environment**			
			Zip code			
			**Childhood home environment**			
			Number of public rooms in home and number of people expected to share sanitation facility			

a *ROI, region of interest*.

b *VBM, voxel-based morphometry*.

c *SBM, surface-based morphometry*.

d *DTI: diffusion tensor imaging*.

e *Income to Needs (ITN), total family income divided by the federal poverty level for a family of that size, in the year data was collected*.

## SES variables reported in structural imaging studies

Although many studies have reported a high degree of correlation between various components of SES, different socioeconomic factors reflect different aspects of experience and should not be used interchangeably (Duncan and Magnuson, 2012). For example, families with greater economic resources may be better able to purchase more nutritious foods, provide more enriched home learning environments, or afford higher-quality child care settings or safer neighborhoods. In contrast, parental education may influence children's development by shaping the quality of parent–child interactions (Duncan and Magnuson, 2012). The notion that these SES components might differentially influence development is supported by the neuroscience literature, in which whole-brain structural analyses (Lange et al., 2010; Jednoróg et al., 2012) and studies with a priori testing of regions of interest (Hanson et al., 2011; Noble et al., 2012a; Luby et al., 2013) have indicated that different SES components may be associated with different brain structural attributes. Additionally, SES disparities tend not to be global, but rather, are disproportionately associated with differences in the structures of the hippocampus, amygdala, and the prefrontal cortex (see Table 1).

### Income

Household or family income is usually calculated as the sum of total income, typically measured monthly or annually. Although income can be considered a continuous variable, many studies ask participants to select what category of income they fall into. For example, a participant may indicate that they earn between $30,000 and $60,000 dollars per year, and researchers often take the midpoint of the participant's estimate (i.e., $45,000), thereby reducing variability between participants. Income is one of the more volatile of the SES markers, as family circumstances frequently fluctuate across time, resulting in varying levels of income throughout childhood and adolescence (Duncan, 1988; Duncan and Magnuson, 2012). **Income-to-Needs** (ITN) is a similar marker of SES, in which total family income is divided by the official poverty threshold for a family of that size. Hanson et al. (2011); Noble et al. (2012a) and Luby et al. (2013) all find significant positive correlations between income/ITN and hippocampal size, with children and adolescents from lower SES families having smaller hippocampal volumes. Examining income-related differences in amygdala volumes, we find some discrepancies across studies. While both Hanson et al. (2011) and Noble et al. (2012a) find no association between income/ITN and amygdala volume, Luby et al. (2013) report a significant positive correlation, where children from lower income homes also have smaller amygdala volumes. The families in the latter study reported lower family income than the families in the other two studies; thus it may be possible that, unlike the hippocampus, substantial income insufficiency is necessary to observe structural differences in amygdala volumes.

KEY CONCEPT 3. Income-to-Needs The ratio of total family income divided by the federal poverty level for a family of that size, in the year data were collected. A family living at the poverty line would have an income-to-needs of ratio of 1. In 2012, 20.4 million people reported an income below 50% of their poverty threshold, including 7.1 million children under the age of 18.

### Education

Parental education or educational attainment is usually measured by participants reporting their highest level (or their parents' highest levels) of education (e.g., college degree). While family income has been associated with resources available to the family and levels of environmental stress (Evans and English, 2002), parental education has been more closely linked to cognitive stimulation in the home (Hoff-Ginsberg and Tardif, 1995). Compared to parents with lower levels of education, parents with higher levels of education tend to spend more time with their children (Guryan et al., 2008), use more varied and complex language (Hart and Risley, 1995; Hoff, 2003), and engage in parenting practices that promote socioemotional development (Duncan et al., 1994; McLoyd, 1997; Bradley and Corwyn, 2002). Again, like income/ITN, we find some inconsistencies across studies when examining links between parental education and children's brain structure. Luby et al. (2013) and Noble et al. (2012a) find no significant correlations between parental education (measured as the average or highest level of education of any parents or guardians living in the home) and hippocampal volumes. Hanson et al. (2011) report a significant association between right hippocampal volumes and paternal, but not maternal, education levels. There are differences across studies in reported amygdala volumes as well. Whereas Noble et al. (2012a) find a negative correlation between parental education and amygdala volumes, Luby et al. (2013) and Hanson et al. (2011) find no association. These differences may be due in part to how parental education was measured (average parental education vs. separate indicators for mothers and fathers) and/or how parental education was coded (continuously vs. categorically).

Examining the relation between brain structure and one's own educational attainment in adulthood (as opposed to parental education), both Gianaros et al. (2012) and Piras et al. (2011) found positive associations between educational attainment and increases in white matter integrity using diffusion tensor imaging (indexed by increases in fractional anisotropy and decreases in mean diffusivity, respectively). Whereas Gianaros and colleagues found widespread associations, Piras and colleagues found that, once controlling for age, only microstructural changes in the hippocampi significantly correlated with educational attainment. Noble et al. (2012b) also found no simple correlation between reported educational attainment and either hippocampal or amygdala volumes in adulthood. Educational attainment did, however, moderate the association between age and hippocampal volume. Specifically, as has been reported previously, age was quadratically related to hippocampal volume, with the volume of this structure tending to increase until approximately the age of 30, at which point volume starts to decline (Grieve et al., 2011). Although this quadratic relation between hippocampal volume and age was present across the entire sample, the volumetric reduction seen at older ages was more pronounced among less educated individuals, and was buffered among more highly educated individuals. Differences in hippocampal structure between higher and lower educated individuals may therefore be most apparent in the later stages of the lifespan.

### Occupation

Occupations generally reflect education, earnings, and prestige (Jencks et al., 1988), and have been extensively studied as an important aspect of SES as they are directly related to both education and income. Chiang et al. (2011) found that occupational status, measured using the Australian Socioeconomic Index (SEI), a 0–100 scale based on an individual's occupational category, was not related to white matter integrity. However, the authors did find an interaction between occupational status and white matter integrity, controlling for subjects' age and sex. Specifically, higher SEI was associated with higher heritability white matter integrity in the thalamus, left middle temporal gyrus, and callosal splenium.

### SES composite measures

Some studies have combined different SES markers to create average or composite measures. Cavanagh et al. (2013) used indicators of early life SES (number of siblings, number of people per room, paternal social class, parental housing tenure, and use of car by family) and current SES (current income, current social class, and current housing tenure) to predict cerebellar gray matter volume. Both composite measures positively predicted cerebellar structure, where current SES explained significant additional variance to early life SES, but not vice-versa. Staff et al. (2012) also measured both childhood SES (indexed by paternal education and childhood home conditions) as well as adult SES (indexed by the individual's educational attainment, occupational status, and neighborhood deprivation). These authors reported a significant association between hippocampal volume and childhood SES, after adjusting for the individual's SES as an adult more than 50 years later. These results may suggest that early life conditions may have an effect on structural brain development over and above conditions later in life.

The Hollingshead scale (Hollingshead, 1975) is a commonly used measure of SES, which combines occupation and education (Two-Factor Index) or occupation, education, marital status, and employment status (Four-Factor Index). Duncan and Magnuson (2003) have argued that aggregating these SES measures is faulty as fluctuations within each measure of SES differentially affect parenting and child developmental outcomes. Imaging studies using these composite measures of SES have found significant correlations between composite scores and regions in the medial temporal lobe and frontal lobe (Raizada et al., 2008; Jednoróg et al., 2012), but without knowing associations to specific SES markers, it is difficult to compare these studies with other structural imaging studies.

### Neighborhood SES

Of note, SES can describe a single participant, the participant's family or even the participant's neighborhood. The neighborhood context is associated with various health outcomes (Pickett and Pearl, 2001) as it is another source of potential exposure to stressors (e.g., violence) or protection from them (e.g., community resources, social support). Some studies have found correlations between neighborhood disadvantage and cognitive outcomes independent of individual level SES (Wight et al., 2006; Sampson et al., 2008), whereas others have not (Hackman et al., 2014). Studies examining neighborhood SES and brain structure have also had mixed findings. Gianaros et al. (2007, 2012) have used census tract level data (median household income, percentage of adults with college degrees or higher, proportion of households below federal poverty line, and single mother households) to create composite indicators of community SES. Although community SES was not associated with total brain volume or gray matter volumes in regions of interest (Gianaros et al., 2007), community SES was positively associated with white matter integrity independent of self-reported levels of stress and depressive symptoms (Gianaros et al., 2012). Similarly, Krishnadas et al. (2013) found that neighborhood SES, indexed using the Scottish Index of Multiple Deprivation, was related to **cortical thickness**, with men living in more disadvantaged areas demonstrating more cortical thinning in areas that support language function (bilateral perisylvian cortices) than men living in more advantaged areas.

KEY CONCEPT 4. Cortical thickness Defined in neuroimaging studies as the shortest distance between the white matter surface and pial gray matter surface.

### Subjective social status

Finally, subjective social status is another marker of SES used in some research. In these studies, participants are typically asked to indicate on a drawing of a ladder where they believe they rank in terms of social standing among a particular group. In past studies, lower social ladder standings have been correlated with negative physical and mental health outcomes (Adler et al., 2000; Kopp et al., 2004; Hu et al., 2005), even after accounting for objective measures of education, income, and potential reporting biases (Adler et al., 1994). Gianaros et al. (2007) found that subjective social status was not correlated with hippocampal or amygdala volumes, but was significantly associated with reduced gray matter volume in the perigenual area of the anterior cingulate cortex (pACC). This finding may be understood by recognizing that the pACC is a region in the brain involved in experiencing emotions and regulating behavioral and physiological reactivity to stress. Measures of subjective social status may not take into account objective measures of SES, but relate more to the individual's experience of disadvantage.

### Words of caution in selecting SES variables

Collecting and utilizing multiple independent measures of SES is necessary to accurately assess structural brain changes throughout development. SES is too complex to be captured by a single indicator or even a composite measure. Each measure of SES is its own distinct construct with varying associations with experience and cognitive development. However, while SES variables are not interchangeable, they are nonetheless highly correlated. It is therefore essential to avoid model multicollinearity in statistical analyses. This may be accomplished by first carefully considering which variables are most appropriate for testing particular hypotheses, and then confirming low variance inflation factors (VIF) within the model. Increasing sample size, centering variables, and utilizing residuals are additional methods to avoid inappropriate analysis and interpretation.

As a final word of caution, many of the SES indicators referenced above are based on studies completed in Western countries. Further work will be necessary to explore the generalizability of findings across different countries and cultures (Minujin et al., 2006; Lipina et al., 2011).

## Covariates, mediators, and moderators

When examining SES disparities in brain structural development, additional demographic factors must be considered as well. First and foremost, the age of the participant must be taken into account, as brain structural volumes change significantly across childhood and adolescence (Paus et al., 1999; Lenroot and Giedd, 2006). Further, the timing of volumetric growth and reductions vary across different brain structures (Grieve et al., 2011). Inconsistencies in results across studies highlighted above may therefore be due to variability in the age ranges of the samples studied. Caution is advised when generalizing results reported within a narrow-age-range sample, as SES disparities in brain structure may vary substantially as a function of age.

Several studies include relatively wide age ranges, recruiting, for example, both children and adolescents in their imaging samples (Lange et al., 2010; Hanson et al., 2011; Noble et al., 2012a; Lawson et al., 2013). Two additional studies have taken a lifespan approach to examining SES and structural brain development (Piras et al., 2011; Noble et al., 2012b). Incorporating wide age ranges into a study allows researchers to consider whether results vary as a function of participant age. For example, both Noble et al. (2012b) and Piras et al. (2011) examine associations between subcortical structures and educational attainment in a wide age range of participants. Piras et al. (2011) found that microstructural changes in the hippocampus, but not changes in gross volume in this structure, were significantly predicted by education levels. However, due to a large negative correlation between education and age, the decreases in microstructure may have been more closely related to older age than greater education. As discussed above, Noble et al. (2012b) reported that higher levels of educational attainment buffered against age-related reductions in hippocampal volume, signifying that the association between age and hippocampal volume is not constant across all levels of education. Of course, distinctions between development and decline are, in some respects, arbitrary, and may be more appropriately classified according to functional rather than structural measures.

Sex is another important demographic characteristic to consider. Volumetric variation in brain structures increase within and between males and females during puberty (Sowell et al., 2003). Sex differences have been reported for cortical thickness. Using a longitudinal sample of participants ages 9–22 years, Raznahan et al. (2010) observed differences in cortical maturation, with males demonstrating a thicker cortex in frontopolar regions at younger ages and subsequent greater cortical thinning than females during adolescence. It has also been reported that females demonstrate more rapid cortical thinning than males in specific cortical areas (right temporal, left temporoparietal junction, and left orbitofrontal cortex) corresponding to the “social brain” (Mutlu et al., 2013). It will be important in future work to better understand how the links between SES variables and structural brain development may vary by sex, and/or a combination of sex and age.

In addition, studies have reported that families living in chronic poverty have differential outcomes based on when and for how long poverty was experienced (National Institute of Child Health and Human Development Early Child Care Research Network, 2005). While the brain is most malleable in early childhood, it nonetheless retains a substantial degree of plasticity throughout the lifespan, and the extent to which the timing and duration of socioeconomic disadvantage are associated with brain structural differences is virtually unexplored in the neuroscience literature to date.

Finally, it is important to consider environmental exposures and experiences that may account for links between distal socioeconomic factors and brain structural differences. For example, Luby et al. (2013) recently reported that links between income and hippocampal volume were mediated by caregiving support/hostility and stressful life events. Of course, there are many potential experiential correlates of SES that have not been well studied in the context of SES disparities in brain development, including nutrition, exposure to environmental toxins, safety of the play environment, or quality of the child's linguistic environment. In order to develop interventions that effectively target the SES gap in achievement, it will be essential to try to understand the particular component(s) of the environment that are most influential in explaining disparities.

## Volume vs. cortical thickness/surface area

Differences in findings across studies may also be accounted for by the techniques used to measure morphometry. Most studies examining SES differences in brain structure have reported **cortical volumes** as their outcome of interest (but see Jednoróg et al., 2012; Liu et al., 2012; Krishnadas et al., 2013; Lawson et al., 2013). However, cortical volume is a composite measure that is determined by the product of **surface area** and cortical thickness, two genetically and phenotypically independent structures (Panizzon et al., 2009; Raznahan et al., 2011). Though the cellular mechanisms are not fully understood, it has been hypothesized that symmetrical cell division in the neural stem cell pool contribute to exponential increase in the number of radial columns that result in surface area, without changes to cortical thickness. In contrast, asymmetrical cell division in founder cells is independently responsible for a linear increase in the number of neurons in the radial column, leading to changes in cortical thickness but not surface area (Rakic, 2009). As such, these two properties of the cortical sheet develop differentially; cortical surface area tends to *expand* through childhood and early adolescence and decrease in adulthood, whereas cortical thickness tends to *decrease* rapidly in childhood and early adolescence, followed by a more gradual thinning and ultimately plateauing (Schnack et al., 2014). Cortical thinning is related to both synaptic pruning and increases in white matter myelination, resulting in a reduction of gray matter as measured on MRI (Sowell et al., 2003). These maturational changes occur concurrently and together contribute to the development of the mature human brain.

KEY CONCEPT 5. Cortical volumes The most commonly used outcome in studies of socioeconomic disparities in brain structure. Cortical volume is actually a composite of cortical thickness and surface area, two genetically and phenotypically distinct morphometric properties of the brain.

KEY CONCEPT 6. Surface area The area of exposed cortical surface or convex hull area (CHA) and the area of cortex hidden in sulci.

Thus, studies in which the dependent measure is cortical volume may not adequately reflect the complexities of morphometric brain development. Indeed, cross-sectional comparisons of cortical volume are poor indicators of brain maturation (Giedd and Rapoport, 2010), whereas cortical thickness has been shown to be a more meaningful index of brain development (Sowell et al., 2004; Paus, 2005) and has been associated with both cognitive ability (Porter et al., 2011) and behavior (Shaw et al., 2011). For example, IQ has been correlated with the trajectory of cortical thickness, such that, during childhood, more intelligent children have thinner cortices than children with lower IQ, with this association strengthening through adolescence. In contrast, by middle adulthood, a thicker cortex is related to higher IQ (Schnack et al., 2014). Importantly, IQ has also been independently correlated with the trajectory of surface area development, such that more intelligent children exhibit greater surface area during childhood, though surface area expansion is completed earlier and then decreases more quickly in more intelligent adults (Schnack et al., 2014). Together, these findings suggest that both surface area and cortical thickness may be critical in accounting for individual differences in cognitive abilities, and that these factors must be considered independently rather than lumping them into a single composite measure of cortical volume.

In summary, when considering associations between experience and brain morphometry, cortical thickness and surface area should be assessed separately, rather than reporting on the composite metric of cortical volume (Winkler et al., 2010; Raznahan et al., 2011). Research investigating cortical complexity and its association with SES variables will be vital to further understanding how environmental influences over the life course influence structural brain development.

## Conclusions

Children living in socioeconomic disadvantage are more likely to experience cognitive delays and emotional problems (Brooks-Gunn and Duncan, 1997), but the underlying causal pathways between disadvantage and developmental outcomes are not clear. The nascent field of socioeconomic disparities in brain structure is an exciting one, which holds promise in helping to understand this question. However, while progress has been made in understanding how socioeconomic disparities may affect brain development, there are many avenues for further research. Careful social science approaches to assessing individual socioeconomic factors must be combined with cutting-edge neuroscientific approaches to measuring precise aspects of brain morphometry. Consideration of how results interact with demographic factors such as age and sex are critical. Differences in exposures and experiences that may mediate socioeconomic disparities in brain development must be rigorously assessed to help identify or confirm underlying mechanisms.

Although this review has focused on SES disparities in brain structure as opposed to function, it is readily acknowledged that the two approaches are complementary. While a structural approach lends itself to greater spatial resolution as well as, arguably, more precision in understanding proximal experience-dependent mechanisms, it is limited in terms of functional interpretations. Ultimately, linking both structural and functional imaging to cognitive outcomes is essential for examining associations between anatomy, physiology, and behavior. Brain structural measures can be viewed as mediators between SES and cognition, or as outcome variables in their own right; having clear theoretical pathways ensures accurate interpretation of results and implications, and will help inform the design of effective policies, emphasizing early and targeted interventions.

### Conflict of interest statement

The authors declare that the research was conducted in the absence of any commercial or financial relationships that could be construed as a potential conflict of interest.
